# Marburg Virus Disease: Epidemiology, Immune Responses, and Innovations in Vaccination and Treatment for Enhanced Public Health Strategies

**DOI:** 10.3390/pathogens14050468

**Published:** 2025-05-12

**Authors:** Tafadzwa Dzinamarira, Claude Mambo Muvunyi

**Affiliations:** 1 ICAP, Columbia University, Lusaka 37417, Zambia; 2 Africa Centre for Inclusive Health Management, Stellenbosch University, Stellenbosch 7600, South Africa; 3 School of Health Systems & Public Health, University of Pretoria, Pretoria 0002, South Africa; 4 Rwanda Biomedical Centre, Kigali P.O Box 7162, Rwanda; claude.muvunyi@rbc.gov.rw

Marburg virus disease (MVD) remains an important global health concern, in part because of its particularly high mortality rate [1,2]. Caused by orthomarburgviruses, members of the *Filoviridae* family, the disease mainly appears in sporadic outbreaks in Sub-Sharan Africa, making the surveillance and response efforts difficult [3]. Nearly 20 outbreaks of MVD have been reported globally, including recent events in Tanzania (June 2023 and January 2025) and Rwanda (November 2024) [4,5]. The recurrence emergency of MVD outbreaks in Ebola-prone regions underscores the complexity of controlling filovirus outbreaks and the need for continued research and robust health system preparedness.

MVD outbreaks are often linked to zoonotic transmission from fruit bats (Rousettus aegyptiacus), the natural reservoir of orthomarburgviruses [6]. Human-to-human transmission typically occurs through direct contact with the bodily fluids of infected individuals or contaminated surfaces. MVD outbreaks tend to occur in remote areas, where healthcare infrastructure is limited, complicating timely detection and response efforts.

The immune response to MVD poses a significant challenge in managing the infection. The severe pathogenesis of *Filoviridae* infections, including MVD, is characterized by widespread viral replication, leading to exceptionally high viral titers in the bloodstream [7]. This extensive replication triggers detrimental host responses, such as excessive cytokine production and the release of tissue factors and other mediators that contribute to the clinical manifestations of severe disease, including liver damage, vascular leakage, and hemorrhaging [8]. These effects are indicative of the virus’s ability to effectively counteract host antiviral defenses, particularly interferon (IFN) responses, which are crucial components of the innate immune response to viral infections [9]. Understanding the immune mechanisms related to MVD is critical for the development of effective vaccines and therapeutic strategies.

Several promising therapeutic approaches towards MVD are being explored. Current research is focused on antiviral drugs targeting viral replication, entry, and assembly, as well as host-targeting therapies aimed at modulating immune responses [9,10]. Small molecule inhibitors, including those that block viral RNA polymerase and viral entry, show potential in preclinical models [10]. RNA interference techniques, such as antisense oligonucleotides, also hold promise in disrupting viral replication [9,10]. Additionally, host-directed antivirals, such as immune modulators like interferons, cytokine/chemokine modulators, and broad-spectrum antivirals, are being considered as adjunctive treatments [10,11]. Another area of focus is the development of therapeutic antibodies and convalescent plasma, which can neutralize the virus and mitigate disease severity in infected individuals [12]. Despite these advances, there are no approved treatments or vaccines for MVD due to the virus’s genetic variability and the challenges of drug resistance and viral escape mechanisms. Notably, the scarcity of clinical investigating MVD therapeutics remains a significant research gap [13]. In addition, the need for combination therapies and rapid diagnostic tools to optimize treatment initiation is critical. Moreover, equitable access to these emerging therapies, alongside strong public health preparedness, will be vital for controlling outbreaks and achieving global health security. Moving forward, continued research is essential to identify safe and effective treatments, address the remaining gaps in vaccine and therapeutic development, and ultimately enhance our ability to manage and prevent Marburg virus outbreaks.

Vaccine clinical trials have shown promise due to the development of experimental vaccines that have demonstrated protective efficacy in animal models [10,14]. However, in regions at risk of MVD outbreaks, challenges remain in vaccine procurement, scaling up distribution, and ensuring adequate surveillance systems to detect outbreaks early. Continued research into vaccines, alongside improved diagnostic tools and public health strategies, is essential for enhancing preparedness and minimizing the global impact of future MVD outbreaks. Despite notable scientific advancements, inherent global health challenges remain, particularly in ensuring equitable access to MVD vaccines and therapeutics. One of the primary barriers is the timely procurement and distribution of this lifesaving prevention and treatment, especially in remote or underserved regions where healthcare infrastructure is limited [15]. Geographic inaccessibility, inadequate cold chain systems, and logistical constraints can significantly delay response times during outbreaks. Furthermore, public perception and vaccine hesitancy remain persistent issues. Misinformation, limited awareness of vaccine safety and efficacy, and mistrust in health authorities can all hinder the successful rollout of vaccination campaigns [16].

To overcome these barriers, public health strategies must be holistic and inclusive. Strengthening risk communication, building community trust, and engaging local leaders and health workers are essential to improve vaccine uptake and adherence to containment measures. The complex nature of MVD outbreaks necessitates cross-sectoral collaboration involving researchers, public health practitioners, governments, non-governmental organizations, and communities. Integrating insights from epidemiology, immunology, and vaccine development into policy and practice will enhance outbreak preparedness and response capabilities. Additionally, national governments and international partners must invest in strengthening health systems, particularly in regions at high risk of filovirus outbreaks. This includes bolstering disease surveillance networks, enhancing laboratory diagnostic capacity, training frontline healthcare workers, and developing rapid response teams capable of deploying during outbreaks. Health system preparedness is crucial for minimizing the morbidity and mortality associated with MVD and for preventing spillover events from escalating into larger public health crises [17].

This Special Issue aims to highlight that addressing MVD requires a multifaceted and sustained effort. We encourage contributions that advance our understanding of the virus and its interaction with hosts, evaluate public health interventions, and propose innovative solutions for outbreak detection, control, and prevention. Through continued research, community engagement, and global cooperation, we can build more resilient systems capable of responding effectively to MVD and future emerging infectious disease threats.
